# First report of canine ocular thelaziosis in the Republic of Moldova

**DOI:** 10.1186/s13071-019-3758-3

**Published:** 2019-10-30

**Authors:** Mirabela Oana Dumitrache, Angela Monica Ionică, Eugeniu Voinițchi, Nicolai Chavdar, Gianluca D’Amico

**Affiliations:** 1 Department of Parasitology and Parasitic Diseases, University of Agricultural Sciences and Veterinary Medicine Cluj-Napoca, Calea Mănăştur 3-5, Cluj-Napoca, 400372 Cluj, Romania; 2 CDS-9, “Regele Mihai I al României” Life Science Institute, University of Agricultural Sciences and Veterinary Medicine Cluj-Napoca, Calea Mănăştur 3-5, Cluj-Napoca, 400372 Cluj, Romania; 3 Faculty of Veterinary Medicine, State Agrarian University of Moldova, 48 Mircești Street, Chișinău, Republic of Moldova; 4 Veterinay Clinic Ciavdar, Nicolae Costin 61, Chișinău, Republic of Moldova

**Keywords:** *Thelazia callipaeda*, Dogs, Vector-borne zoonosis, Republic of Moldova

## Abstract

**Background:**

Countries of eastern Europe are considered, due to several risk factors, more vulnerable to infections with newly (re)emerging pathogens. During the last decade, in several European countries, reports of autochthonous cases of ocular thelaziosis due to *Thelazia callipaeda* have been published, posing a great concern from both veterinary and public health perspective. However, in the Republic of Moldova only limited epidemiological data are available regarding zoonotic vector-borne pathogens and, until now, no data exist on the zoonotic nematode *T. callipaeda.*

**Methods:**

In September 2018, an 11-year-old dog, mixed-breed, intact male was referred to a private veterinary clinic from Chișinău, Republic of Moldova, with a history of 2 weeks of an ocular condition affecting the right eye. The ophthalmological exam revealed the presence of nematode parasites in the conjunctival sac and under the third eyelid. The collected parasites were identified by morphological techniques and molecular analysis.

**Results:**

A total of 7 nematodes were collected, and 5 females and 2 males of *T. callipaeda* were identified morphologically. The BLAST analysis confirmed the low genetic variability of this parasite in Europe. The travel history of the patient allowed us to confirm the autochthonous character of the case.

**Conclusions:**

To the best of our knowledge, this is the first report of thelaziosis in dogs from the Republic of Moldova, which confirms the spreading trend of *T. callipaeda* and the existence of an autochthonous transmission cycle of this zoonotic parasite in the country.
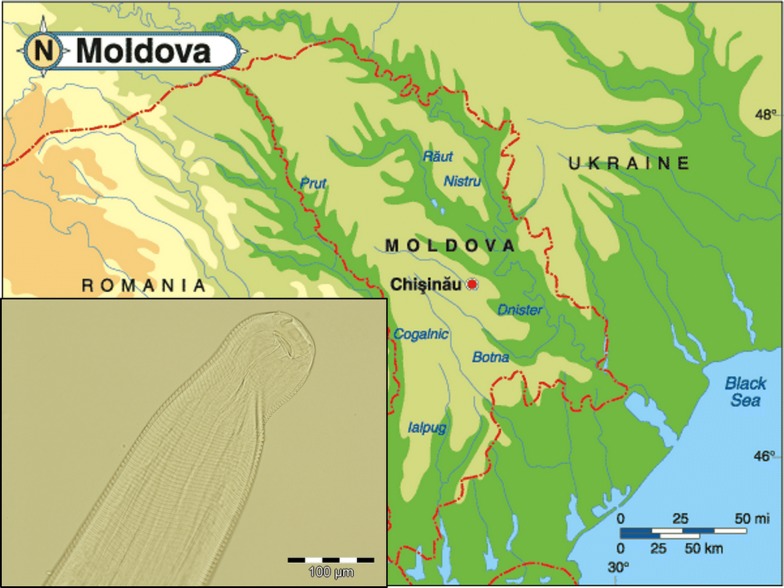

## Background

Canine vector-borne diseases (CVBDs) of zoonotic concern consist in a group of illnesses affecting both human and canine populations. They are caused by aetiological agents of variable pathogenicity and are transmitted by arthropod vectors. CVBDs rapid spreading worldwide in areas considered previously as non-endemic or with sporadic cases, the important role of dogs in the modern society involving a close and long-lasting contact with humans, and the favourable environmental conditions for development of vectors have led to an increasing veterinary and public health interest in these diseases [1]. Different favouring factors such as climate change, urbanization, land use, etc., have increased the exposure risk for both dogs and humans in many areas worldwide [1].

However, despite the high general interest and number of studies conducted to assess CVBD’s epidemiology, the mechanisms involved in these diseases transmission and clinical expression, and the complex role of asymptomatic dogs are not completely understood [1, 2]. This is mainly due to insufficient data on vectors and pathogens distribution, and the lack of recent and updated surveillance studies [1]. Among CVBDs, ocular thelaziosis is regarded as having a moderate zoonotic relevance [1]. In Europe and Asia, the sole aetiological agent, *Thelazia callipaeda*, is a parasite localised in the conjunctival sacs, naso-lacrimal ducts, under the eyelids and the nictitating membrane in different species of mammals, predominantly in domestic and wild carnivores, and also in humans [3, 4]. This nematode is responsible for ocular symptoms of variable severity: epiphora, local pruritus and congestion, photophobia, mild to severe conjunctivitis and keratitis, which can evolve to cornea opacification and even ulceration in the absence of diagnosis and therapy [4, 5].

Although until 2005 the complete life-cycle of this parasite was not known, nowadays, the biology is elucidated in both the intermediate and definitive hosts [5–7]. *Thelazia callipaeda* is transmitted by male *Phortica variegata* (Drosophilidae) that feed on the definitive hosts’ lachrymal secretions. In the lachrymal secretions of parasitized mammals, first-stage larvae (L1) are released by adult females after mating. During the feeding process, the flies become infected with L1, which undergo a series of transformations and become infective L3 stage in the body of the intermediate host. L3 larvae are inoculated to the receptive host during the feeding process, and consecutively develop to adults [4, 7]. The parasite was known for decades as the “oriental eye worm” due to its distribution limited to different territories of Asia and the former Soviet Union [3]. In Europe, *T. callipaeda* was first reported in dogs in Italy [8]. Regarded initially as a “new” agent of an ocular condition in various species of domestic and wild mammals, and also in humans, it is now considered endemic in many regions of different European countries such as Spain, Portugal, Italy, France and Switzerland [9–13]. However, in the last decade, in many other countries (Croatia, Romania, Hungary, Bosnia and Herzegovina) previously considered outside of the distribution area, autochthonous cases of ocular thelaziosis caused by *T. callipaeda* in dogs were reported, highlighting an eastern spreading of this parasite [14]. Moreover, in the same time frame, reports in other host species (e.g. cats, wild cats, mustelids, golden jackals, foxes, lagomorphs) have been published [12, 15]. It is now clear that there is a positive relation between animal and human cases. In areas where the disease is highly prevalent in pets (cats and dogs) and wild carnivores (especially in foxes, that seem to play an important role in the epidemiology of *T. callipaeda*), human cases may occur [5, 14]. In different countries of eastern Europe, the socio-economic instability of the last three decades, the low awareness of veterinarians and physicians on newly (re)emerging pathogens and the high density of rural, poor communities, are considered as important risk factors for neglected diseases. Infection with *T. callipaeda* is considered as one of these diseases [14]. Moreover, there is a lack in scientific data regarding vector-borne pathogens’ epidemiology in these geographical areas.

This study presents the first autochthonous case of ocular thelaziosis in a dog in the Republic of Moldova and identifies the haplotype circulating in this country, thus extending the knowledge on the epidemiology of *T. callipaeda* in Europe.

## Methods

In September 2018, an 11-year-old dog, mixed-breed, intact male, with a history of ocular condition affecting the right eye for a period of 2 weeks was referred to a private veterinary clinic from Chișinău, the capital city located in the centre of the Republic of Moldova. The dog was born and had lived its entire life in a private yard from a neighbourhood located in north-western Chișinău (47°02′03″N, 28°47′17″E).

A general and an ophthalmological consultation were performed. Physical examination revealed a good condition of the animal. Due to the excessive ocular discharge and blepharospasms, the ophthalmic examination of the right eye was possible only after the administration of a local anaesthetic. The close examination of this eye revealed the presence of translucent and mobile parasites in the conjunctival sac and under the third eyelid. All worms were mechanically removed from the affected eye, by using sterile, blunt tweezers and by flushing of the conjunctival sac with saline solution (0.9% NaCl). The dog was treated by application of a single dose spot-on formulation containing imidacloprid 10% and moxidectin 2.5%. The collected parasites were preserved in tubes with formalin or absolute ethanol for further investigations. The owner provided the travel history of the dog and verbally consented to the usage of the collected material for scientific purpose.

To perform the morphological identification, the specimens stored in formalin were mounted on a glass slide. Microscopic examination was performed using an Olympus BX61 with an adapted DP72 camera. Morphological keys and descriptions available in the literature were used for the parasite identification [16]. The genomic DNA was extracted from specimens (2 females) preserved in absolute ethanol, by using a commercial kit (Isolate II Genomic DNA Kit, Bioline, London, UK) following the manufacturer’s instructions. The DNA samples were further processed by PCR amplification of a 670-bp fragment of the *cox*1 gene using the NTF/NTR primer pair, as previously described [17]. The amplicons were sequenced using an external service (performed at Macrogen Europe, Amsterdam, NL) and the attained sequences were compared to those available in the GenBank database by Basic Local Alignment Search Tool (BLAST) analysis.

## Results

**Fig. 1:**
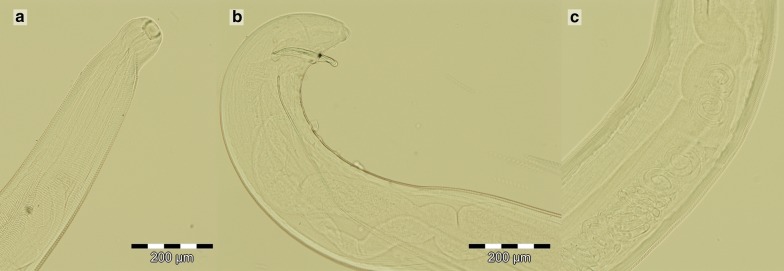
**a** Anterior extremity of recovered worms: a serrated cuticle with transverse striations and a buccal capsule; **b** Male, posterior extremity with pre- and post-cloacal papillae and two unequal spicules; **c** Gravid female, with the vulvar opening situated anterior to the esophago-intestinal junction, and the proximal end of the uterus containing L1 larvae

Following the ophthalmological examination, seven nematodes were collected from the right eye; close examination of the eye revealed conjunctivitis, epiphora, periocular alopecia and erosions. The left eye presented no lesions and no parasites were detected. According to the owner, the dog never travelled abroad. All collected nematodes were examined by light microscopy. All parasites presented specific features, which allowed sex and species identification based on published morphological identification keys [16]. Five females and two males of *T. callipaeda* were identified. The anterior extremity of the recovered worms presented a serrated cuticle with transverse striations (Fig. 1a). The sex was assigned based on several characteristics; *T. callipaeda* males were characterised by a curved posterior extremity, with pre- and post-cloacal papillae and two unequal spicules (Fig. 1b); females of *T. callipaeda* presented the vulvar opening situated anterior to the oesophago-intestinal junction, and the proximal end of the uterus contained L1 larvae (Fig. 1c).

The BLAST analysis of the two sequences revealed a 100% nucleotide similarity to a sequence of *T. callipaeda* haplotype h1 (GenBank: AM042549) in both cases. The sequence was deposited in the GenBank database under the accession number MN163032.

## Discussion

The present study represents the first report of canine ocular thelaziosis caused by *T. callipaeda* in the Republic of Moldova. Reported initially as sporadic, this disease has rapidly become endemic in different countries of the Mediterranean Basin (i.e. Spain, Portugal, Italy and France) and western Europe (Switzerland). Recently, many other countries from eastern Europe (Croatia, Romania, Hungary and Bosnia and Herzegovina) reported “first autochthonous cases” of canine thelaziosis, confirming the north-eastern spreading of this parasites. So far, the eastern limit of the distribution area of *T. callipaeda* in Europe was defined by various reports from Greece [18], Bulgaria, Romania [14, 19] and Slovakia [20]. Considering the latest expanding trend and the recent reports from the bordering countries (Romania and Slovakia), the following cases should have been expected from Ukraine and/or Republic of Moldova, where, to the best of our knowledge, the parasite has not been reported yet.

In this frame, the present report confirms the expected spread of this nematode and establishes a new eastern distribution limit in Europe. This finding is of particular importance due to its autochthonous character. Given that the dog had never travelled outside the country and the Republic of Moldova is considered as a suitable location for the development of *P. variegata* [21], the proven vector of this parasite [6, 7], is an important proof that sustain the existence of an autochthonous transmission cycle of the zoonotic parasite *T. callipaeda* in the Republic of Moldova. Dogs serve as sentinels for human infection in case of different CVBDs [22], including ocular thelaziosis [5]. The close relationship between the parasites’ biology in dogs, other animal hosts (both wild and domestic) and humans is sustained by the occurrence of human cases in areas where the infection in animals is established and also by the low genetic variability of *T. callipaeda* in Europe [5]. Although in Asia other 21 haplotypes have been identified [23], so far only the haplotype h1 has been identified in Europe, regardless the host species [23, 24].

The BLAST analysis of our sequences revealed a 100% similarity to a sequence of *T. callipaeda* haplotype h1, extending the knowledge on this nematode to other territories, where no data were available. This finding also strengthens the hypothesis that in Europe, the spread of the parasite was generated by a single introduction event followed by numerous transmissions [24]. The relatively rapid dissemination of *T. callipaeda* was facilitated by its high adaptability for survival [24] and by circulation of companion animals, especially dogs, between the European countries [21]. For instance, it is suspected that canine *T. callipaeda* was initially introduced in Spain through hunting dogs from Italy and/or travelling companion animals from France, both countries being endemic at that time [25]. Moreover, the first case of thelaziosis in the UK was reported in a dog imported from Romania (neighbouring country of the Republic of Moldova). The same study presented two other cases in patients (dogs) with recent history of travel in endemic countries (Italy and France) [26]. All pets, mainly dogs originating in areas were the disease has been reported, may act as sources of *T. callipaeda*, and by travels or importations can pose a risk for canine and human population in their non-endemic destinations [26].

In most countries (i.e. Italy, France, Hungary, Romania, Slovakia) where *T. callipaeda* is now present, the history of ocular thelaziosis started with case reports in dogs, that were shortly followed by other reports in various species and/or by epidemiological survey that highlighted a more extensive character of the disease. In the absence of any scientific data regarding thelaziosis in the Republic of Moldova, we can only presume that this report is the first of those to come. Our finding highlights the need for additional large-scale studies that should provide information on the current situation of thelaziosis in this country. In many countries where the disease has been reported, it is believed that the information related to this parasite gained in the veterinary field are more consistent and refined compared with those available in human medicine [5], stressing out the importance of the One Health approach. It is now clear that only by providing updated epidemiological data that can increase the awareness of both veterinarians and physicians, the prevention and the early diagnosis of the diseases are possible.

## Conclusions

To the best of our knowledge, this is the first report of autochthons thelaziosis in dogs from the Republic of Moldova. This study extends the current geographical distribution of *T. callipaeda*, confirms the low genetic variability of the nematode in Europe and highlights the importance of thelaziosis in the differential diagnosis of ocular conditions in both animals and humans from the Republic of Moldova.

## Data Availability

All data generated or analysed during this study are included in this published article.

## Abbreviations

CVBD: canine vector-borne disease
BLAST: Basic Local Alignment Search Tool
